# Evaluation of the Management and Outcome of Patients with Retained Products of Conception after Gestational Week 23+0: A Retrospective Cohort Study

**DOI:** 10.3390/jcm13154439

**Published:** 2024-07-29

**Authors:** Petra Pateisky, Fanny Mikula, Marija Adamovic, Jana Neumüller, Kinga Chalubinski, Veronica Falcone, Stephanie Springer

**Affiliations:** Department of Obstetrics and Gynecology, Division of Obstetrics and Feto-Maternal Medicine, Medical University of Vienna, 1090 Vienna, Austria; petra.pateisky@meduniwien.ac.at (P.P.); fanny.mikula@meduniwien.ac.at (F.M.); marija_adamovic@gmx.at (M.A.); j.m.neumueller@gmail.com (J.N.); kinga.chalubinski@meduniwien.ac.at (K.C.); veronica.falcone@meduniwien.ac.at (V.F.)

**Keywords:** residual trophoblastic tissue, retained products of conception, RPOC, placenta accreta spectrum, PAS, ultrasound, conservative management, surgical management

## Abstract

**Background:** Retained products of conception after childbirth or miscarriage are associated with an increased rate of maternal complications, such as abnormal vaginal bleeding and infections. Late complications may also include intrauterine adhesions, causing infertility. Surgical interventions carry a certain risk. Thus, conservative management is often discussed as an alternative. The aim of this study was to assess the clinical outcomes of patients with retained products of conception, comparing a primary surgical approach to conservative management. **Methods:** We conducted a retrospective cohort study of 88 patients diagnosed with retained products of conception after 23+0 weeks of gestation at the Medical University Vienna between 2014 and 2022. **Results:** Forty-seven (53.4%) patients underwent primary surgical management and 41 (46.6%) primary conservative management. After primary conservative treatment, a complication could be observed in 10 (24.4%) women. In contrast, complications occurred in 32 (68.1%) women in the group with primary surgical treatment (*p* < 0.001). The most common complication in both groups was the ongoing suspicion of retained products of conception. Patients after primary surgical treatment were significantly more likely to require a secondary change in treatment (*p* < 0.001). Ultimately, secondary conservative management was applied in 30 (63.8%) patients. In contrast, only nine (21.95%) patients with primary conservative management required secondary surgical management. **Conclusions:** Due to the high risk of complications and persistent retained products of conception, primary surgical management should only be prioritized in hemodynamically instable or septic patients.

## 1. Introduction

After pregnancy, whether following a term delivery, a preterm delivery or a miscarriage, retained products of conception (RPOC) remaining in the uterus, also called residual trophoblastic tissue, can lead to maternal complications such as infection or abnormal vaginal bleeding. These complications are associated with substantial maternal morbidity [1]. Furthermore, residual trophoblastic tissue may lead to long-term complications, such as endometritis and intrauterine adhesions (IUAs), potentially causing infertility [2,3]. Although the exact incidence is unknown, RPOC affects, regardless of the delivery mode, about 1% of term pregnancies [4]. After preterm birth (PTB) and miscarriage or the termination of pregnancy (TOP) in the first or second trimester, a higher prevalence of up to 6% can be observed. RPOC occurs even more frequently after medical abortions, with a prevalence of up to 15% [2].

The risk factors for placental retention or RPOC are a previous pregnancy with placental retention, a PTB or miscarriage in the second trimester, uterine malformations, a previous uterine surgery (curettage, cesarean section, myoma surgery), preeclampsia, intrauterine fetal death, fetal growth restriction, placental abnormalities, velamentous cord insertion, a maternal age ≥ 30 years and a placenta accreta spectrum (PAS) [5,6,7]. Patients with RPOC clinically often present with abnormal vaginal bleeding or discharge, fever, amenorrhea and/or pelvic pain [8,9,10]. Due to the variable clinical and sonographic presentation and lack of defined universal diagnostic criteria, diagnosis can be challenging. Usually, the sonographic diagnosis is based on the presence of an intrauterine hyperechogenic focal mass, with a poorly defined endometrium–myometrium interface, a fluid layer and/or an increased and irregular endometrial thickness [11,12]. An additional color Doppler assessment may improve the diagnostic precision [1,13,14].

Due to the wide range of symptoms that RPOC can present, and as it can occur from an early gestational age to term pregnancy, no generally accepted management is recommended. On the one hand, a primary surgical procedure may be necessary in cases of life-threatening bleeding, hemodynamic instability and/or pending sepsis. On the other hand, unnecessary surgical management can induce significant bleeding, thereby requiring a hysterectomy [15]. Further, injury of the decidua basalis and adhesions may be induced (30% after D&C), potentially leading to infertility, miscarriage or abnormal placentation [16,17]

According to multiple studies, the conservative management of RPOC seems to be effective [3,6]. However, the term RPOC includes a broad spectrum of possible causative clinical scenarios. Thus, the population of patients with RPOC benefitting the most from an expectant management approach is still not clear. Surgical interventions such as dilatation and curettage (D&C) or transcervical resection (TCR) have been shown to be efficient tools for RPOC management [7]. Still, there is the known significant pending risk of inducing severe hemorrhage requiring a hysterectomy [4]. Hence, alternative treatment options such as a conservative approach warrant closer evaluation. In a retrospective observational cohort study by Takahashi et al. on 59 patients with RPOC, 39% (23 patients) could be treated effectively and conservatively, resulting in the resolution of the RPOC [18]. The risk stratification concerning severe bleeding complications with possible ultrasound markers for optimal patient selection is being investigated currently [19,20,21].

Thus, the careful assessment for the benefits of a conservative approach versus the risk of profound bleeding complications still needs to be elaborated. Therefore, we evaluated the outcomes of RPOC in patients after 23 weeks of pregnancy. The aim of our retrospective study was to compare the effectiveness and outcomes between the primary conservative versus the primary surgical management of RPOC at a single center over the last 9 years.

## 2. Materials and Methods

This single-center retrospective cohort study was executed at the Department of Obstetrics and Gynecology, Division of Obstetrics and Feto-Maternal Medicine, a tertiary referral center at the Medical University of Vienna, Austria. Medical data from all patients who were treated due to suspected RPOC after 23+0 weeks of gestation at our center between 2014 and 2022 were extracted from our database. Data were acquired by using the Viewpoint^®^ software (Version 5.6.28.56, GE-Viewpoint Healthcare, Wessling, Germany). Clinical records were evaluated for data on maternal, fetal, pregnancy, neonatal and postnatal outcomes.

Both singleton and twin pregnancies following birth or the termination of pregnancy (TOP) were included in this study. Patients with a delivery or a TOP before 23+0 weeks of gestation were excluded from this study. Overall, we included 354 women with suspected RCOP in the study. In total, 117 (33.1%) patients matched the inclusion criteria and 29 (8.2%) pregnancies were lost to follow-up. Thus, 88 (24.9%) patients were evaluated in this analysis. The main outcome was to evaluate the differences in the complication rate between a primary conservative and a primary surgical approach in patients with suspected RPOC. Particular attention was paid to maternal blood loss, the administration of red blood cell concentrates, the occurrence of infections, the need for a hysterectomy or maternal admission to an intensive care unit and the time to the resolution of RPOC.

Gestational age was based on first-trimester ultrasound screening, whenever available, or on the first dating scan recorded. A preterm birth (PTB) was defined as delivery before 37+0 gestational weeks. All patients managed at our center with clinical symptoms after any form of delivery (spontaneous abortion or medically induced abortion, vaginal delivery or vacuum delivery or cesarean section) suggestive of RPOC routinely receive a transvaginal and/or transabdominal scan for evaluation before hospital discharge. These ultrasound examinations are usually performed by two experienced doctors. In the case of acute, severe symptoms (such as massive bleeding, clinical instability, suspicion of incomplete placenta right after delivery, etc.), the ultrasound is performed immediately (often still within the delivery room) by the present staff. The initial available ultrasound evaluation of every patient included within this study was taken as the starting point for the diagnosis and initial management plan.

The decision regarding the first management approach was primarily based upon the severity of the eventual present symptoms in combination with the ultrasound findings and the clinical status of the patient. If these together did not require an intervention based upon clinical judgement, both options were discussed with the patient, and, based on clinical recommendation, the further treatment plan was decided. We classified the different groups of postpartum hemorrhage according to the four clinically known categories of possible underlying pathology following the definitions for each category according to the guidelines of the WHO for postpartum hemorrhage (Guideline on Prevention and Management of PPH, WHO). The estimated blood loss after delivery was retrieved from the medical records based upon one or more of the following (whichever was available): weighing all used gauzes, towels or other cloths/drapery for the collection/absorption of blood (routinely performed in our center in the case of suspected higher blood loss) and/or the blood volume from suction drains if applicable or available. In cases where these measures were not available, the estimated blood loss was based upon the clinician’s overall judgement/estimate in the clinical scenario. The volume of retained products of conception on ultrasound was assessed by measuring the longitudinal, transversal and anterior–posterior maximum dimensions of the suspected RPOC and calculating the volume from these measurements (see example ultrasound pictures for RPOC in the Supplementary Materials Figure S1). Primary conservative management was defined as patients having RPOC managed with medication addressing uterine contractility or expectant management or only receiving the manual removal of the placenta (MROP) immediately after delivery, without additional surgical procedures, followed by observation and/or further medical treatment. Primary surgical management was defined as the need for any surgical intervention due to the suspicion of RPOC, such as the manual removal of the placenta plus additional suction or dilatation and curettage or other surgery up to hysterectomy. A change in management (secondary conservative or surgical management) was defined as a switch from the primarily planned management strategy. All cases of suspected ongoing abnormalities on ultrasound, such as still significant remnants of RPOC, were followed until the lesions disappeared or only minimal remnants were present.

The study was conducted according to the guidelines of the Declaration of Helsinki and approved by the Institutional Review Board of the Medical University of Vienna (protocol code 1729/2022 and date of approval 1 December 2022). Pseudonymized data from medical records were used; thus, patient participation was not necessary. Nevertheless, following the internal hospital standard operating procedure, a written declaration of consent was obtained at registration regarding data use for scientific purposes.

### Statistical Analysis

Statistical analysis was performed with SPSS version 29.0.0.0 for Windows (IBM SPSS Inc., Armonk, NY, USA). Statistical methods involved descriptive statistics, reported the as mean (±standard deviation) for normally distributed continuous variables and median (interquartile range) for non-normally distributed continuous variables. A Kolmogorov–Smirnov test was performed to identify non-normally distributed continuous variables. In the case of continuous variables, the two groups were compared using Student’s t-test or the Mann–Whitney U test, as appropriate. Categorical variables were compared with the chi-square test or Fisher’s exact test. To calculate correlations, Pearson’s correlation was used. Differences were considered statistically significant if the *p*-values ≤ 0.05.

## 3. Results

During the study period, overall, 22,921 pregnant women delivered at our hospital. Out of 354 women with suspected RPOC according to the inclusion criteria, 117 (33.1%) matched the inclusion criteria, while 29 (8.2%) pregnancies were lost to follow-up. Thus, 88 (24.9%) patients were evaluated in this analysis. Overall, 17 (19.3%) patients had twin pregnancies and 71 (80.7%) singleton pregnancies. Of the 88 patients, 47 (53.4%) underwent primary surgical management and 41 (46.6%) primary conservative management. From the primary surgically managed patients, in 33 patients, histology confirmed RPOC (70.2%); in 12 patients (25.5%), no histology result was available; and in two patients (4.3%), histology did not confirm RPOC. The basic characteristics are listed in Table 1. The basic characteristics of both groups significantly differed only in the case of the delivery mode. Patients with primary conservative management had significantly more often a cesarean compared to women with primary surgical management (*p* = 0.009).

The presence of PAS was suspected in 14 (15.9%) patients. Patients with suspected PAS had significantly more D&C in their history (*p* = 0.045). Otherwise, no significant differences could be associated with the presence of PAS.

A total of 44 (50%) patients had a postpartum hemorrhage. This included 28 (59.6%) patients with primary surgical management and 16 (39%) with primary conservative management (*p* = 0.09—not quite reaching statistical significance). The two groups showed a statistically significant difference in the administration of drugs for uterine contraction, being higher in the surgical intervention group (*p* = 0.002). Interestingly, the noted estimated blood loss did not differ significantly (600 mL in the primary conservative management group versus 575 mL in the primary surgical management group). Nevertheless, a correlation between the endometrial width (0.28; *p* = 0.02) as well as the volume of RPOC (0.34; *p* = 0.006) and the estimated blood loss could be demonstrated. In addition, there was a significant correlation between the endometrial width (0.34; *p* = 0.003) and the number of erythrocyte concentrates applied. Further differences in patients with primary conservative management and primary surgical management are listed in Table 2.

In the group of primary surgically managed patients, RPOC was identified significantly earlier (*p* < 0.001). The median time was 0 (0–12) days, compared to 13 (3–39) days in patients with primary conservative management. The diagnosis was made significantly more often while still hospitalized (*p* < 0.001). Further, the resolution time of RPOC was significantly shorter in the group of primary surgically managed patients (*p* = 0.038), with a median time of resolution of 35 days, versus 69 days in patients with primary conservative management. Interestingly, the volume of RPOC did not correlate with the time until resolution (0.081; *p* = 0.54).

Regarding the symptoms leading to the suspicion of RPOC, the two groups only differed in the diagnosis of an incomplete placenta. An incomplete placenta was diagnosed in 17 (39%) patients with primary surgical management, compared to four (9.8%) patients in the other group (*p* = 0.05).

After primary conservative treatment, a complication was observed in 10 (24.4%) women. In contrast, complications occurred in 32 (68.1%) women in the group with primary surgical treatment. These differences were statistically significant (*p* < 0.001). The outcomes of patients after primary management are listed in Table 3. The most common complication in both groups was the ongoing suspicion of RPOC. Patients in the primary surgical group were significantly more often affected (65.96% vs. 21.95%, *p* < 0.001). A total of 39 (44.3%) patients required a secondary change in management. Patients after primary surgical treatment were significantly more likely to require a secondary change in treatment (*p* < 0.001). Ultimately, conservative management was continued in 30 (63.8%) patients. In contrast, only nine (21.95%) patients with primary conservative management required secondary surgical management.

Of the nine initially conservatively managed patients who required secondary surgical management (eight D&C and one hysterectomy), none required further surgical intervention. Nevertheless, RPOC was still suspected in four (44.4%) patients. In the group of patients with secondary conservative management, of the 30 patients, seven women (23.3%) had further complications. Further, five of the 30 secondary conservatively managed patients ultimately required further surgery (three D&C and two hysterectomies). The outcomes of patients after secondary management are listed in Table 4.

## 4. Discussion

Overall, from the total study cohort of 88 patients with RPOC, 47 (53.4%) underwent a primary surgical management and 41 (46.6%) a primary conservative management approach. Patients with a primary conservative management approach had significantly more often a cesarean compared to women with primary surgical management (53.7% versus 25.5%, *p* = 0.009). RPOC was identified significantly earlier (*p* < 0.001) in the primary surgical management group, with the median time being 0 (0–12) days postpartum, compared to 13 (3–39) days in the primary conservative approach. The only significantly different symptom of RPOC in our patient cohort was the suspicion of an incomplete placenta, being present in 17 patients (36.2%) in the surgical management group versus four patients (9.8%) in the conservative management group (*p* = 0.05), concurrent with the dominance of PPH in the former. Our study clearly demonstrated higher complication rates after a primary surgical treatment strategy, with complications being observed in 10 women (24.4%) after a primarily conservative treatment, versus 32 (68.1%) women in the surgical management group (*p* < 0.001). Only nine (21.95%) patients with primary conservative management required secondary surgical management, in comparison to 30 patients (63.8%) in the primary surgical treatment arm requiring further close observation.

The significant difference in the delivery modes between the two management strategies is in contrast to other data, although the literature for direct comparison is scarce (if available at all). A possible explanation for our findings may be the fact that, after a vaginal delivery and/or late pregnancy termination, in the case of eventual risk factors present for RPOC or increased peripartum vaginal bleeding, special focus is placed on checking the integrity of the placenta, including ultrasound [2,22]. In contrast to our data, a prospective observational study on 41 patients with secondary postpartum hemorrhage and RPOC found no statistically different correlation between the mode of delivery and success of primary medical treatment [23]. In this study, roughly two thirds of the patients needed additional surgical treatment; notably, the symptoms of RPOC for inclusion in the study differed from ours.

The time point of the first evaluation of the patient’s symptoms regarding possible RPOC differs within the already published literature, as well as the symptoms taken into account, further complicating the applicability and comparability of the results. In contrast to the data of Schulte et al., where patients were included upon emergency department presentation with secondary PPH and evidence of RPOC, in our analysis, the suspicion of RPOC was based on a broader spectrum of clinical and/or sonographic symptoms with varying time points of first diagnosis [23]. In our study, RPOC was identified significantly earlier (*p* < 0.001) in the primary surgical management group, with the median time being 0 (0–11) days postpartum, compared to 13 (3–39) days in the primary conservative approach. Thus, the diagnosis in the first group was made significantly more often while still hospitalized (*p* < 0.001), possibly reflecting the slightly higher urgency.

The only significantly different symptom in our patient cohort was the suspicion of an incomplete placenta, being present in 17 patients (36.2%) in the surgical management group versus four patients (9.8%) in the conservative management group (*p* = 0.05), concurrent with the dominance of PPH in the former. These findings are in line with previously published data highlighting the higher need for surgical management in patients presenting with primary PPH/severe bleeding [24,25]. Also, in the retrospective analysis of Kobayashi et al., in 96 patients with RPOC-ARC (RPOC and related conditions), a statistically significant difference regarding blood loss at delivery (1330 mL (586–4556) versus 320 mL (24–4654) (*p* = 0.004)) was seen between patients needing invasive procedures (n = 9) and those managed without these interventions (n = 88) [19]. Still, the procedures applied differed compared to other studies (including ours), as uterine artery embolization (UAE) was frequently used.

In accordance with the above-mentioned findings, 28 (59.6%) patients with primary surgical management and 16 (39%) with primary conservative management had a postpartum hemorrhage (PPH), although this finding did not reach statistical significance (*p* = 0.09). Possibly, the suspicion of higher blood loss was the reason for performing a primary surgical approach instead. Although the estimated blood loss did not differ significantly between the two groups, drugs for the improvement of uterine contractility were significantly more often applied in the primary surgical management group (87.2%) versus the primary conservative management group (56.1%). Unfortunately, we could not assess the amount of blood loss in patients that presented with the suspicion of RPOC after initial discharge from the hospital, as this information was not systematically available. Still, our data are in line with the principal findings of the prospective study by Fox et al. with respect to the higher need for surgical management in the presence of suspected postpartum hemorrhage and/or an incomplete placenta.

Although surgical management seems necessary in quite a substantial amount of patients, the associated risks should be considered.

Specifically, the risk of PAS after any form of (intra-)uterine intervention has been shown to be substantially elevated [26,27,28]. In accordance with these data, the 14 patients (15.9%) in our cohort with suspicion of PAS showed significantly more D&C in their histories (*p* = 0.045). Aside from this, our study clearly demonstrated higher complication rates after a primary surgical treatment strategy, with complications being observed in 10 women (24.4%) after a primarily conservative treatment, versus in 32 (68.1%) patients in the surgical management group. These differences were highly statistically significant (*p* < 0.001). As the patients after primary surgical treatment in our study were significantly more likely to require a secondary change in treatment (*p* < 0.001) as well, this finding warrants special attention.

In both groups, the ongoing suspicion of RPOC was the major complication after primary management, with 31 patients from the surgical group being significantly more often affected, versus nine patients conservatively managed (65.96% vs. 21.95%, *p* < 0.001). Only nine (21.95%) patients with primary conservative management required secondary surgical management, in comparison to 30 patients (63.8%) in the primary surgical treatment arm requiring further close observation. In those nine patients with secondary surgical management, eight women received D&C and one woman needed a hysterectomy; after this, no further surgical intervention was required. In contrast, in the group secondarily receiving conservative treatment, seven women (23.3%) of 30 developed further complications requiring another surgery, resulting in three D&C and two hysterectomies, corresponding to a rate of a secondary needed surgical intervention of 16.7%. Thus, overall, the ongoing suspicion of RPOC was higher after secondary surgical management (four patients, 44.4%); however, none of them needed any further intervention. We consider this finding especially interesting as it is known that, in particular, multiple surgical uterine procedures increase the risk for adverse sequelae [7]. Previous dilation and curettage has been shown to be an independent risk factor for a non-adherent retained placenta (OR 12.80, 95%CI 10.57–15.50) in a subsequent vaginal delivery [29].

To date, no generally accepted consensus on a standardized method for the postpartum ultrasound diagnosis of RPOC has been agreed upon [30]. The management of RPOC is further complicated by the fact that highly vascular RPOC can be mistaken for acquired uterine arteriovenous malformations (AVMs) [7]. From our whole study cohort, a total of 44 (50%) patients had a postpartum hemorrhage, without taking specifically into account special vascularization indices. A correlation between the endometrial width (0.28; *p* = 0.02) or the volume of RPOC (0.34; *p* = 0.006) and the estimated blood loss could be demonstrated. This is in line with the results from Schulte et al. in their observational study on the medical management of RPOC, showing that a greater endometrial thickness on ultrasound was significantly associated with a requirement for secondary surgical intervention (*p* < 0.05). Thus, these findings indicate that simple greyscale ultrasound features may enable patient selection for allocation to optimal treatment strategies without the need to thoroughly characterize the vascularity [19].

Our study represents the management of patients with RPOC in the second half of pregnancy at a tertiary referral center with a focus on the possibility of conservative management. However, the retrospective study design may have several limitations with respect to the applicability of the results to other patient cohorts. First, it is possible that not all relevant information could be retrieved regarding decision-making for the chosen management approach. Second, histopathological confirmation was not universally possible, an unavoidable limitation, since many of the patients were treated conservatively or no histology reports were available. The decision regarding the optimal primary treatment may have been biased by the subjective impression of urgency perceived by the different clinicians. Further, there is the possibility of the inaccurate diagnosis of RPOC on ultrasound. There was also no randomization between the two treatment strategies possible. Consequently, this may have caused the differences in the delivery modes between the two groups, with significantly more cesarean sections in the primary conservative management group, potentially indicating different clinical awareness depending on the initial delivery mode.

A further potential limitation is that we could not assess the possible impact of different vascularization patterns on the decision-making. Not all patients had the same length of follow-up; thus, only in those with a few months of postpartum follow-up, a statement can be made about full resolution. We can state that most patients treated for RPOC at our institution had no further symptoms after about 3 months’ time. Further, for their initial evaluation, most patients were seen by one of two experienced doctors for the sonographic examination and the following recommendation for treatment. Still, some patients were seen by other colleagues, directly followed by an intervention in the case of emergency presentation, possibly presenting a bias in the interpretation of the ultrasound findings.

Our study emphasizes that patients managed conservatively with RPOC have overall good outcomes and are at a lower risk of experiencing complications. Moreover, the patients in the surgical management group significantly more often failed the initial treatment approach, needing secondary interventions, and such patients are at a higher risk for adverse maternal outcomes (e.g., performance of a hysterectomy).

## 5. Conclusions

Our study suggests that a conservative management approach may be feasible in hemodynamically stable patients, minimizing the risk of additional complications due to surgery. The occurrence of PPH differed significantly between the two patient cohorts, being significantly higher in the primary surgically managed patients (59.6% versus 39%). The suspicion of an incomplete placenta was the symptom significantly more often noted in the primary surgical intervention group (36.2%) versus the primary conservative intervention group (9.8%). Primary surgical management led to significantly more complications than conservative management (68.1% versus 24.4%), with the ongoing suspicion of RPOC being the main problem (65.96% versus 21.95%). Most importantly, from the initially surgically managed patients, overall, five patients needed a second surgical intervention, corresponding to a rate of secondary surgical intervention of 16.7%.

## Figures and Tables

**Table 1:** Basic characteristics of 88 pregnancies with suspected RPOC.

Variable	Primary Conservative Management (*n* = 41)	Primary Surgical Management (*n* = 47)	*p*-Value
Nulliparity ^1^	24 (58.5)	24 (51.1)	0.53
Maternal age (years) ^2^	34 (31–37)	34 (29–38)	0.72
Body mass index ^2^	22.3 (20.7–25.0)	22.6 (20.6–25.2)	0.88
ART ^1^	9 (22)	10 (21.3)	1.0
Smoking ^1^	4 (9.8)	6 (12.8)	0.75
Termination of pregnancy ^1^	2 (4.9)	0 (0)	0.5
Gestational age at delivery ^2^	38 (34–39.6)	38.3 (35.7–40.1)	0.53
Preterm delivery < 37+0 ^1^	17 (41.5)	11 (23.4)	0.11
Cesarean delivery ^1^ History of uterus surgery ^1^ History of cesarean ^1^ History of D&C ^1^	22 (53.7) 17 (41.5) 5 (12.2) 12 (29.3)	12 (25.5) 14 (29.8) 2 (4.3) 11 (23.4)	0.009 0.27 0.24 0.63
Suspected PAS ^1^	6 (14.6)	8 (17.0)	1.0
Prenatally ^3^	5 (83.3)	2 (25.0)	-
Postnatally ^3^	1 (16.7)	6 (75.0)	-
Accreta ^3^	3 (50.0)	6 (75.0)	-
Increta ^3^	3 (50)	2 (25.0)	-
Percreta ^3^	0 (0)	0 (0)	-

^1^ Number (percent), chi-quadrat test; ^2^ median (interquartile range), Mann–Whitney U test, ^3^ number (percent calculated in relation to the number of patients with suspected PAS). ART, assisted reproductive technology; D&C, dilation and curettage; PAS, placenta accreta spectrum.

**Table 2:** Differences in patients with primary conservative management and primary surgical management.

Variable	Primary Conservative Management (*n* = 41)	Primary Surgical Management (*n* = 47)	*p*-Value
Postpartum hemorrhage ^1^	16 (39.0)	28 (59.6)	0.09
Tonus ^3^	4 (25.0)	11 (39.3)	0.35
Tissue ^3^	11 (68.8)	24 (85.7)	0.1
Trauma ^3^	2 (12.5)	2 (7.1)	1.0
Thrombin ^3^	0 (0)	0 (0)	-
Estimated blood loss after delivery ^2^	600 (400–1000)	575 (350–1000)	0.922
Administration of erythrocyte concentrates ^1^	15 (36.6)	21 (44.7)	0.52
Administration of other blood products ^1^	2 (4.9)	8 (17.0)	0.1
Time until suspicion of RPOC (days) ^2^	13 (3–39)	0 (0–12)	<0.001
Time until resolution of RPOC (days) ^2^	69 (43–89)	35 (16–81)	0.038
Endometrial width (mm) ^2^	25 (20–31)	25 (21–35)	0.48
Volume RPOC (ccm) ^2^	26.5 (8.6–59.2)	32.2 (18.2–62.6)	0.68
Diagnosis during hospitalization	20 (48.8)	40 (85.1)	<0.001
Drug therapy (Oxytocin, Misoprostol, Methylergometrine)	23 (56.1)	41 (87.2)	0.002
Symptoms of RPOC			
Routine sonography ^1^	15 (36.6)	9 (19.2)	0.093
Bleeding ^1^	20 (48.8)	27 (57.5)	0.521
Fever ^1^	3 (7.3)	3 (6.4)	1.0
Endometritis ^1^	2 (4.9)	2 (4.3)	1.0
Retained lochia ^1^	0 (0)	1 (2.1)	1.0
Abdominal pain ^1^	2 (4.9)	3 (6.4)	1.0
Incomplete placenta ^1^	4 (9.8)	17 (36.2)	0.05
Intraoperative diagnosis ^1^	1 (2.4)	7 (14.9)	0.06

^1^ Number (percent), chi-quadrat test; ^2^ median (interquartile range), Mann–Whitney U test; ^3^ number (percent calculated in relation to the number of patients with postpartum hemorrhage), chi-quadrat test. RPOC, retained products of conception; mm, millimeter; ccm, cubic centimeter. Some women had more than one sign of RPOC.

**Table 3:** Outcomes after primary management.

Variable	Primary Conservative Management (*n* = 41)	Primary Surgical Management (*n* = 47)	*p*-Value
ICU admission	1 (2.4)	2 (4.3)	1.0
Maternal death	0 (0)	0 (0)	-
Complication of primary management ^1^	10 (24.4)	32 (68.1)	<0.001
Bleeding	5 (12.2)	9 (19.2)	0.4
Perforation	0 (0)	2 (4.3)	0.5
Endometritis	2 (4.9)	1 (2.1)	0.6
Ongoing suspicion of RPOC	9 (21.95)	31 (65.96)	<0.001
Hysterectomy	0 (0)	0 (0)	-
Secondary change in management	9 (21.95)	30 (63.8)	<0.001

^1^ Number (percent), chi-quadrat test; Mann–Whitney U test. ICU, intensive care unit; RPOC, retained products of conception. Some women had more than one complication.

**Table 4:** Outcomes of 39 patients with secondary change in management.

Variable	Secondary Conservative Management (*n* = 30)	Secondary Surgical Management (*n* = 9)	*p*-Value
Complication of secondary management ^1^	7 (23.3)	4 (44,4)	0.24
Bleeding ^1^	5 (16.7)	1 (11.1)	1.0
Perforation ^1^	0 (0)	0 (0)	-
Endometritis ^1^	1 (3.3)	0 (0)	1.0
Ongoing suspicion of RPOC ^1^	6 (20.0)	4 (44.4)	0.197
Hysterectomy ^1^	2 (6.7)	0 (0)	
D&C ^1^	3 (10.0)	0 (0)	

^1^ Number (percent); RPOC, retained products of conception; D&C, dilation and curettage. Some women had more than one complication.

## Data Availability

The data presented in this study are available on request from the corresponding author. The data are not publicly available due to local data protection guidelines.

## Supplementary Materials

The following supporting information can be downloaded at: https://www.mdpi.com/article/10.3390/jcm13154439/s1, Figure S1: Example ultrasound pictures of 3 cases with RPOC managed primarily conservatively.
