# Supplementary material for: Evaluation of the Management and Outcome of Patients with Retained Products of Conception after Gestational Week 23+0: A Retrospective Cohort Study
Source: J Clin Med. 2024 Jul 29;13(15):4439. doi: 10.3390/jcm13154439 (PMC11312729; doi:10.3390/jcm13154439)
Supplement: Supplementary file 1 [file jcm-13-04439-s001.zip › jcm-3060272-supplementary.pdf]

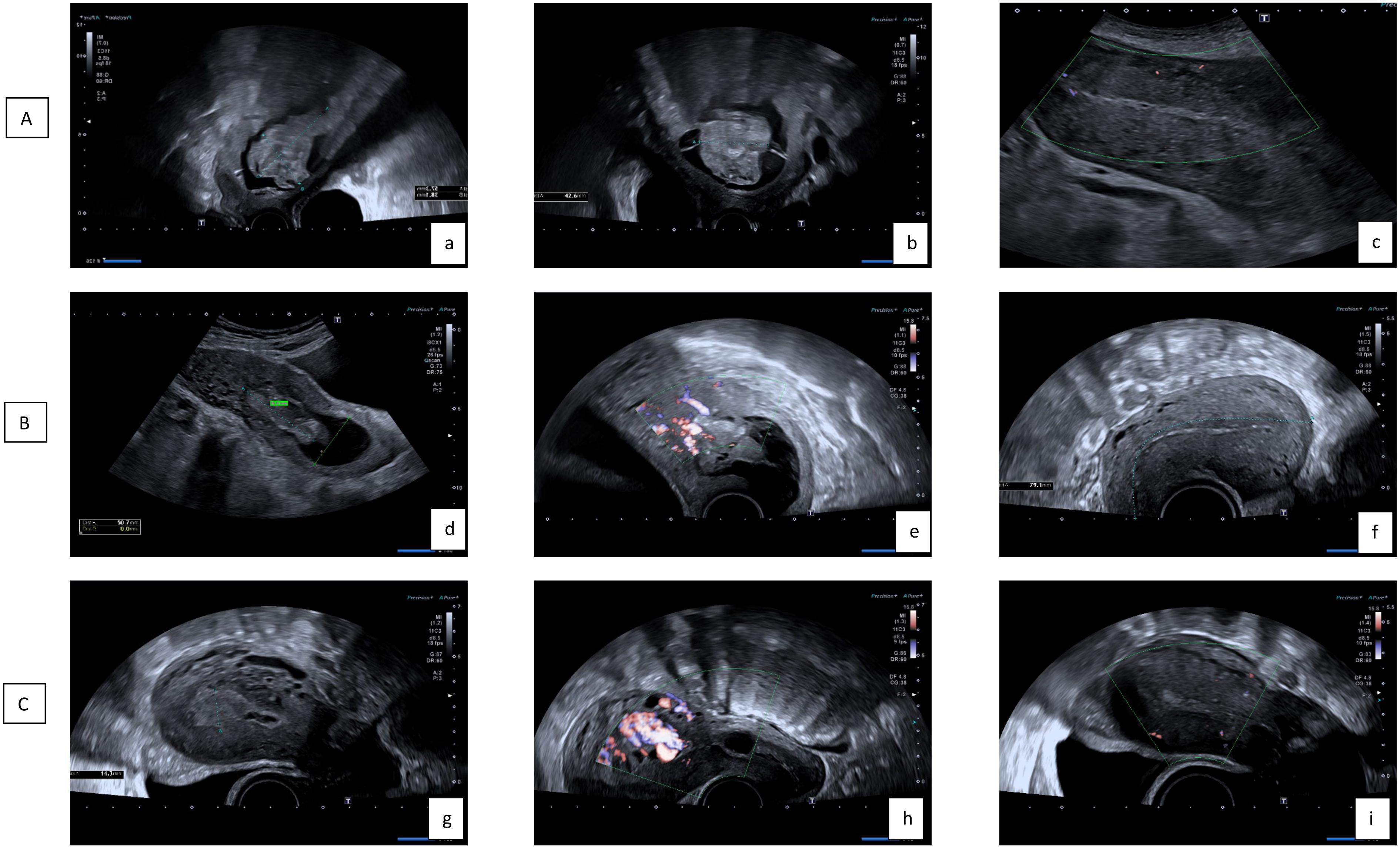
A
B
C
a
b
c
d
e
f
g
h
i

Figure S1. Example ultrasound pictures of 3 cases with RPOC managed primary conservative, rows show the same patient, the earlier two scans are depicted on the left, the final scan on the right; (A) Patient with intracavitary clot and hematometra (a) transvaginal longitudinal (b) transvaginal transverse (c) transabdominal: complete resolution of RPOC (2 days between scans); (B) Patient with adherent placental remnant (d) transabdominal longitudinal: hematometra caudal of RPOC (e) transvaginal Color Doppler: vascular pedicle reaching into the placental remnant (f) transvaginal: complete resolution of RPOC 38 days between scans) ; (C) Patient with increte placental remnant (g) transvaginal longitudinal (h) transvaginal longitudinal with TDI-Color Doppler: pronounced vascularization, RPOC in the myometrium (i) transvaginal: complete resolution of RPOC (67 days between scans)
